# The Prevalence of Dietary Supplements That Claim Estrogen-like Effects in Japanese Women

**DOI:** 10.3390/nu14214509

**Published:** 2022-10-26

**Authors:** Tsuyoshi Chiba, Yuko Tousen, Chiharu Nishijima, Keizo Umegaki

**Affiliations:** 1 Department of Food Function and Labeling, National Institute of Health and Nutrition, National Institutes of Biomedical Innovation, Health and Nutrition, Tokyo 162-8363, Japan; 2 Department of Food Safety and Management, Showa Women’s University, Tokyo 154-8533, Japan

**Keywords:** dietary supplements, *Pueraria mirifica*, estrogen, adverse events

## Abstract

Recently, adverse events, such as irregular vaginal bleeding and menstrual disorders, associated with the use of dietary supplements containing *Pueraria mirifica*, have been reported in Japan. *P. mirifica* contains phytoestrogens, such as deoxymiroestrol and miroestrol. Therefore, we investigated the use of supplements that claim to have estrogen-like effects (i.e., estrogen-like supplements) in Japanese women aged from 15 to 69 years old in an online survey. The prevalence of estrogen-like supplement use was 5%, accounting for approximately 15% of the sample, including ex-users. The majority of the users were in their 40s and 50s, mainly using these supplements for the treatment of menopausal symptoms. In contrast, the younger generation mainly used them for beauty purposes, such as weight loss, mastogenic effects, and skin care. Many of them visited a clinic or took medicines for menstrual-related troubles. In all age groups, soybeans/isoflavones were the most commonly used, followed by equol and placenta. Participants in their teens and 20s also used *P. mirifica*. Among them, 16.2% had experienced adverse events, including irregular vaginal bleeding, breast swelling and pain, and heavy menstruation. In conclusion, estrogen-like supplement use is associated with adverse events; thus, it is necessary to pay attention to the use of these supplement. Furthermore, because the purpose of use differs depending on generation, caution according to each generation is necessary.

## 1. Introduction

Dietary supplements are used for a wide range of purposes other than nutritional supplementation, and the purpose differs depending on the user’s characteristics, such as gender, age, and health condition. It has been reported that the prevalence of dietary supplement use is higher in women compared with men [1,2]. Specifically, women are more likely than men to use dietary supplements for weight loss and beauty purposes [3]. Recently, many products targeting women that claim a mastogenic effect via an estrogen-like action are commercially available on the market. One of the major ingredients of these products in Japan is *Pueraria mirifica*, which contains phytoestrogen, such as deoxymiroestrol and miroestrol. Phytoestrogens are plant-derived compounds that have a similar structure to estradiol and have weak affinity to the estrogen receptor [4]. However, it has been reported that the estrogenic activity of deoxymiroestrol and miroestrol is almost the same as that of 17beta-estradiol [5]; therefore, products containing *P. mirifica* might possess estrogen-like effects. Indeed, the effects of *P. mirifica* on the reproductive organs, bones, cardiovascular diseases, and other symptoms related to estrogen deficiency in menopausal women have been studied [6]. In contrast, there have been a series of reports of adverse events related to the use of products containing *P. mirifica* among young women in Japan, and the National Consumer Affairs Center of Japan cautions consumers on *P. mirifica* [7,8]. This caution from the National Consumer Affairs Center of Japan is mainly for young women, but some products containing *P. mirifica* claim efficacy against menopausal symptoms, meaning that middle-aged and older women might also use these products. In fact, adverse events have been observed in a clinical trial using *P. mirifica* in peri-menopausal women [9].

Post-menopausal women exhibit decreased levels of female hormone secretion, making the estrogenic effects of these supplements attractive. In addition to *P. mirifica*, other supplements that claim estrogen-like effects are used for the purpose of alleviating symptoms of menopausal disorders. In Japan, isoflavones derived from soybeans, which have estrogenic effects, are the most popular ingredients taken by such women, because Japanese people are familiar with soy products, such as tofu, miso, and natto [10,11]. Numerous studies have been conducted on the benefits of soy-based isoflavone intake; a systematic review revealed that an increased soy isoflavone intake increases bone mineral density [12], reduces the risk of breast cancer in pre- and post-menopausal women [13], and decreases the prevalence of menopausal symptoms such as hot flashes and vaginal dryness [14,15]. However, soy isoflavone intake has been shown not to affect other menopausal symptoms, such as vaginal atrophy index and night sweats [15,16]. In addition, around 200 million people worldwide [17], and 12.8 million people in Japan [18], are affected by osteoporosis. The association between menopause and osteoporosis and the role of estrogen in maintain bone mineral density are well known [19]. Estrogen treatment is standard for preventing osteoporosis [20], but there are concerns that prolonged estrogen treatment in postmenopausal women may increase the risk of ovarian cancer [21,22]. In this regard, peri-menopausal women prefer to use supplements that claim estrogen-like effects to prevent osteoporosis.

Compared with young people, more middle-aged and older aged people prefer dietary supplementation. In addition, a significant proportion of people in this population take medicines that include estrogen preparations [23,24], and polypharmacy is a major concern among these populations [25]. In this regard, these generations should be aware of not only the dietary supplement itself, but also the interaction between dietary supplements and drugs. In particular, patients receiving hormone-replacement therapy for menopausal disorders should be concerned about the interaction between hormones and dietary supplements [26]. If dietary supplements contain ingredients that have estrogen-like effects, additive or synergistic effects may be induced in patients undergoing hormone-replacement therapy. However, the status of the use of estrogen-like supplements in women has not been clarified. In this study, we focused on supplements claiming to have female hormone-like effects (i.e., estrogen-like supplements), and investigated the use of estrogen-like supplements by women in each age group, as well as any adverse events.

## 2. Materials and Methods

### 2.1. Participants and Procedures

An online cross-sectional questionnaire survey was conducted by Cross Marketing Inc. (Tokyo, Japan) from 24 September to 2 October 2019. This study was conducted with the approval of the Research Ethics Committee of the National Institutes of Biomedical Innovation, Health and Nutrition (No. 194-2, approved on 18 July 2019), Showa Women’s University (No. 19-15, approved on 21 June 2019), and in accordance with the Declaration of Helsinki. The questions were presented to the research company, who then conducted an Internet survey, collected the survey results, and anonymized the personal information to deliver data which could not be identified. Females from 15 to 69 years of age were registered with the research company as survey monitors. An email with a survey cooperation request and a webpage link to the survey was sent to computer-randomized monitors. An explanation of this study was provided at the beginning of the survey page, and only those individuals who agreed to participate answered the questionnaire. Complete responses were collected on a first come, first served basis, until the numbers reached the quotas for age. A preliminary survey was conducted to elucidate estrogen-like supplement usage and gestational and menopause conditions. Then, the actual survey was conducted on 1200 estrogen-like supplement users from the preliminary survey by age group. The actual survey contained questions about the purpose of dietary supplement use, the major ingredients of dietary supplements, and the experience of adverse events. The questionnaire in this survey is presented as Supplementary Materials.

### 2.2. Statistical Analysis

Differences in the distribution among age groups were compared using a chi-squared (*χ*^2^) test using IBM SPSS Statistics ver. 28.0.1.0 (IBM Corporation, Armonk, NY, USA). A *p*-value of <0.05 was considered statistically significant. Estrogen-like supplement usage is influenced by consumers’ status (age, gestational condition, and menopause condition). These factors were entered into a bivariate logistic regression, and candidate variables were entered into a multivariable logistic regression model. To identify factors of the prevalence of estrogen-like supplement usage, multivariable logistic regression was conducted, and odds ratios (ORs) and 95% confidence intervals (95% CIs) were calculated using EZR ver. 4.0.3 (Saitama, Japan), which is a modified version of R commander designed to add statistical functions frequently used in biostatistics.

## 3. Results

### 3.1. Characteristics of the Participants

To study estrogen-like supplement users, a preliminary survey was conducted on 61,021 females aged 15–69 years (Table 1). Regarding gestational condition, 2.8% were pregnant and 4.0% were lactating. In terms of menopause, 1.7% were before the first menstruation, 66.4% were premenopausal, and 31.9% were post-menopausal.

### 3.2. The Prevalence of Dietary Supplement Use

The prevalence of dietary supplement use is shown in Table 2. Regarding any type of dietary supplement, 28.9% of the participants were currently using dietary supplements, whereas 18.8% used to use dietary supplements. In terms of estrogen-like supplements, 5.0% of the participants were currently using estrogen-like supplements, accounting for 10.5% of the dietary supplement users. Additionally, 2.8% of the participants had used estrogen-like supplements within the last year, accounting for 5.8% of the dietary supplement users. Then, the 1200 participants who were using or had used estrogen-like supplements progressed to the actual survey (Table 1). The characteristics of the participants in the actual survey were almost the same as those in the preliminary survey.

### 3.3. Factors Associated with Estrogen-like Supplement Use

Estrogen-like supplement use might be associated with one’s hormonal condition. Thus, we conducted a logistic regression analysis to identify the factors influencing estrogen-like supplement usage (Table 3). No multicollinearity among the independent variables was suggested by the correlation analysis. In this survey, age, gestational condition, menstruation, menstruation-related problems, dedication for menstruation-related problems, and subjective symptoms suggestive of menopause were identified as significant factors. Among these factors, only menstruation suppressed estrogen-like supplement usage.

### 3.4. Health Conditions among Estrogen-like Supplement Users

Data on health conditions in terms of menstruation are shown in Table 4. Only 19.2% of the premenopausal participants did not have any problem with menstruation, whereas the other 80.8% expressed some problems, such as feeling irritable, depressed, or sleepy before menstruation (46.6%), irregular cycles (42.4%), and severe pain during menstruation (42.3%). In this situation, 11.3% of them were seeing a doctor and 20.8% of them were taking medicine for menstrual-related symptoms (Table 5). In addition, 10.0% of them had subjective symptoms suggestive of menopause, and they were visiting a clinic, whereas 29.2% of them had subjective symptoms suggestive of menopause, but they were not visiting a clinic (Table 6).

### 3.5. The Purpose of Estrogen-like Supplement Use

The purpose of estrogen-like supplement use is shown in Table 7. The main purpose was “Anti-aging (36.8%)”, which was higher in older generations compared with younger generations. Other key purposes were “Treatment for menopause symptom (33.5%)”, “Skin care (31.0%)”, and “Treatment for menstruation-related symptoms (24.5%)”, and “Weight loss (20.0%)”. Except for “Treatment of disease”, there were significant differences among generations. “Weight loss”, “Mastogenic effect”, “Skin care”, and “Treatment for menstruation-related symptoms” were higher in younger generations; on the other hand, “Treatment for menopause symptoms”, “Prevention of diseases”, and “Anti-aging” were higher in older generations.

### 3.6. The Major Ingredient of Estrogen-like Supplements

The main ingredients of estrogen-like supplements which the participants used are shown in Table 8. The most popular ingredient was soybeans/isoflavones (43.3%). The second most popular ingredient was equol (24.4%), which is a metabolite of daidzein by enterobacterium. The third most popular ingredient was the placenta (22.7%), and the prevalence of these three ingredients was higher in older generations compared with younger generations. The other ingredients comprised less than 10%, although the prevalence of *P. mirifica* was 5.2% and was higher in the younger generations compared with the older generations.

### 3.7. Statement of Estrogen-like Supplement Use

In terms of frequency, most of the participants used estrogen-like supplements for five to seven days a week (69.6%), followed by three to four days a week (12.2%), and one to two days a week (9.8%) (Table 9). Regarding duration, most of the participants had used estrogen-like supplements for more than one year (34.3%), followed by one to three months (17.8%) and three to six months (14.3%) (Table 10).

### 3.8. Information Sources and Ways to Obtain Estrogen-like Supplements

The information sources pertaining to estrogen-like supplements are shown in Table 11. The most popular information source was the Internet (58.5%), then television or radio (26.8%), then family, friends, or acquaintances (19.8%). There were significant differences among generations in information sources, but only SNS showed a clear tendency, in that it was higher in the younger generations and decreased age-dependently. According to information source, most obtained estrogen-like supplements via the Internet (57.5%), followed by a pharmacy or drug store (44.2%) and by mail order (9.9%) (Table 12). There was a tendency toward more of the younger generations obtaining estrogen-like supplements at physical stores, such as a pharmacy or drug store, clinic, or supermarket/convenience store compared with the older generations.

### 3.9. Adverse Events

Adverse events that might be associated with estrogen-like dietary supplement use are shown in Table 13. The major adverse event was nausea/vomiting (4.7%), followed by diarrhea/constipation (3.6%) and breast swelling and pain (2.6%). We also asked about supplement use after having experienced adverse events. When they had experienced palpitation/shortness of breath, irregular vaginal bleeding, or eczema/itching, more than half of subjects stopped immediately. However, when they had experienced breast swelling and pain, heavy menstruation, and hot flashes, a lot of them kept using the supplements without changing or decreasing the amount or frequency.

## 4. Discussion

In 2017, a lot of adverse events associated with use of supplements containing *P. mirifica* were reported to the National Consumer Affairs Center of Japan [7]. The major adverse events included irregular menstruation and vaginal bleeding, potentially caused by the estrogenic effects of *P. mirifica*. In addition to *P. mirifica*, there are several other estrogen-like supplements on the Japanese market, resulting in a risk of adverse events if consumers use them inappropriately. To clarify this issue, we conducted an Internet survey regarding estrogen-like supplement usage. In this study, the prevalence of estrogen-like supplement use was 5%, accounting for approximately 15% of the total sample, including ex-users; the most popular ingredients were soybeans/isoflavones. Among them, 16.3% of the participants had experienced adverse events, including symptoms mainly related to physiological effects peculiar to women, such as irregular vaginal bleeding.

We clarified that the prevalence of estrogen-like supplement usage was associated with age, menstruation and menopause conditions, and related problems. Under-menstruation (premenopausal) was a negative factor of estrogen-like supplement use (OR = 0.75, 95% CI = 0.64–0.86); in other words, menopause was one of the factors for estrogen-like supplement usage. In addition, menstruation-related problems (OR = 1.40, 95% CI = 1.24–1.58) and menopause symptoms (OR = 2.54, 95% CI = 2.34–2.75) were factors of estrogen-like supplement use. Consistently, more than 30% of those aged between their teens and 40s used such supplements for the treatment for menstruation-related symptoms, and more than 50% of those in their 50s–60s used them for the treatment of menopause symptoms. This trend could be explained by the age at onset of menopause. It has been reported that the average age of menopause is 48.3 years old in Japan [27] and 51 years old in the USA [28], and 75% of these women are annoyed by symptoms such as hot flashes and night sweats [28]. Post-menopausal women desire to alleviate these symptoms, such as hot flashes, sleep problems, mood disorders, sexual dysfunction, weight gain, and declines in cognitive functioning, and hormone-replacement therapy is an effective treatment for such problematic symptoms. However, there are potentially undesirable health consequences of hormone-replacement therapy in terms of cardiovascular health and the risk of ovarian and breast cancer [29,30]; thus, a lot of post-menopausal women desire to alleviate these symptoms and seek out complementary and alternative medicine, including phytoestrogens [15,31,32,33,34].

In addition, numerous participants used estrogen-like supplements for weight loss and mastogenic effects. In the case of weight loss, 20.0% of the participants, especially those of the younger generations (33.8% in their 10s; 34.5% in their 20s), used estrogen-like supplements for this purpose (Table 7). At this time, there is limited evidence of the efficacy of phytoestrogens on body weight. It has been reported that soy products decrease the body weight, BMI, and body fat ratio in overweight or obese Asian populations [35]. In contrast, however, one systematic review showed no association between phytoestrogen supplementation (e.g., soy isoflavones, daidzein, red clover isoflavones, and flaxseed extract) and body weight, body mass index, waist-to-hip circumference, total fat mass, or percentage of body fat in post-menopausal women [36]. In this regard, there is not enough evidence to encourage the use of estrogen-like supplements for this purpose. In the case of mastogenic effect, 15.7% of the participants (40.0% in their 10s; 37.4% in their 20s) used estrogen-like supplements for this purpose. *P. mirifica* is the most popular ingredient for this purpose in Japan. *P. mirifica* contains a lot of phytoestrogens; miroestrol and deoxymiroestrol are known to be responsible ingredients due to their estrogenic activity [5]. Even though the estrogenic activities of miroestrol and deoxymiroestrol are almost same as those of 17beta-strradiol, quality control has not been conducted on products containing *P. mirifica* which claim mastogenic effects. This situation has caused adverse events in many young women. In light of this, the Japanese government has specified *P. mirifica* as a “Designated Ingredient, etc.” [37]. However, various *P. mirifica* products with attractive claims for women are still on the market, and adverse events are constantly being reported [38].

Soybeans/isoflavones were the most popular ingredients in this survey; there are several studies about the efficacy of soybeans/isoflavones via estrogenic activity on not only menopausal symptoms, but also breast cancer, prostate cancer, cardiovascular diseases, and osteoporosis [39]. In addition, it is reported that isoflavones combination with probiotics improved bone mineral density in postmenopausal women [40]. Prebiotics not only affect the balance of the intestinal flora—this alteration increases the absorption of minerals such as calcium and magnesium, and influences inflammation and the immune system, affecting bone metabolism [41]—but also increase the bioavailability of isoflavones [40]. Probiotics and isoflavones might coordinately prevent osteoporosis. Equol was the second most popular ingredient in this survey. Equol is a metabolite of daidzein which is a soybean isoflavone, and it is produced through intestinal bacterial metabolism [42,43]. There are many reports about the beneficial characteristics of equol products. However, it is also known that approximately 50% of Asians and 25% of non-Asians host the intestinal bacteria that convert daidzein into equol [44]. In addition to soy isoflavones, the market for equol products as supplements has expanded, and studies about equol intake are ongoing. Beneficial effects of equol intake on hot flashes in postmenopausal women have been reported [45], but there is little scientific evidence of the beneficial effects of equol, other than on menopausal symptoms.

We previously reported that patients use dietary supplements in combination with medicines for the purpose of treating diseases, among other reasons [23,46]. The interaction between dietary supplements and medicines is an important issue among patients. In this survey, 20.8% of the participants were taking medicine for menstruation-related symptoms, and these participants were potentially taking medicines that include estrogen preparations [23,24]. In this situation, pharmacodynamic interactions should be taken into consideration. In contrast, 16.4% of the participants were taking medicine for purposes other than menstruation-related symptoms. In this situation, pharmacokinetic interactions should be considered. It has been reported that soybeans/isoflavones [47], equol [48], black cohosh [49], and other herbal ingredients of dietary supplements have the potential to interact with drugs via the modification of drug metabolism [50,51]; their concomitant use can cause serious adverse events [52]. In this survey, we did not ask what kind of medicines the participants were taking; thus, we cannot speculate on the exact risks to our participants. However, we should consider the possibility that some users of estrogen-like supplements may be deterred from appropriate medical care and appropriate self-care.

The strength of this study is that it is the first report clarifying the perceptions of estrogen-like supplement use. In addition, this survey was conducted on 61,021 participants and 1200 estrogen-like supplement users in each generation. In contrast, a limitation of this study is that it was an online survey; thus, the participants were registrants of the survey company. In this regard, we have to carefully treat our data as general, even though Internet and online questionnaires have become popular across all age groups. In addition, 20.8% of the estrogen supplement users were taking medicine for menstruation-related symptoms, and 16.4% of the estrogen supplement users were taking medicine for reasons other than menstruation-related symptoms. However, we did not ask what kind of medicines they were taking, so we could not examine the risk of the adverse events by concomitant use. In addition, the participants were limited to females only. Although phytoestrogens exert health benefits for not only women, but also men, there is not enough evidence of their efficacy and safety or their potential to cause adverse effects [53]. Recently, the demand for estrogen-like supplements among men [54] and trans people [55] has also increased; thus, we should clarify the situation among these populations.

## 5. Conclusions

In this study, the use of supplements with estrogen-like effects was observed in all age groups, and it was clarified that the purpose of use differed according to age group. In addition, health hazards, particularly symptoms attributed to female hormone-like effects, are observed at a certain rate, and the possibility of interactions between estrogen-like supplements and medicines is suspected. Thus, it is necessary to pay attention to the use of these products. The purpose of use differs depending on age; therefore, it is considered necessary to provide information according to each age.

## Figures and Tables

**Table 1:** Characteristics of the participants.

	Preliminary Survey		Actual Survey	
	*n*	%	*n*	%
Total	61,021		1200	
**Age**				
10s ^1^	3997	6.6	130	10.8
20s	9610	15.7	203	16.9
30s	12,930	21.2	217	18.1
40s	14,830	24.3	217	18.1
50s	12,866	21.1	217	18.1
60s	6788	11.1	216	18.0
**Gestational condition**				
None	56,860	93.2	1118	93.2
Pregnant	1726	2.8	44	3.7
Lactating	2435	4.0	38	3.2
**Menopause condition**				
In 30s	846	1.5	24	2.1
In 40s	5304	9.3	93	8.3
In 50s	11,363	20.0	284	25.4
In 60s	648	1.1	18	1.6
Premenopausal	37,751	66.4	686	61.4
Before first menstruation	948	1.7	13	1.2

^1^ 10s, aged from 15 to 19 years old.

**Table 2:** The prevalence of dietary supplement use.

	*n*	%	% ^3^
**Any dietary supplements ^1^**			
I am currently using it	17,658	28.9	
I used to use it, but not now	11,495	18.8	
I have never used it	31,868	52.2	
**Estrogen-like supplements** **^2^**			
I am currently using it	3057	5.0	10.5
I used to use it within the last year	1704	2.8	5.8
I used to use it over a year ago	4135	6.8	14.2
I have never used it	20,257	85.4 ^4^	69.5

^1^ *n* = 61,021; ^2^ *n* = 29,153, including dietary supplement users and ex-users; ^3^ ratio of dietary supplement users to ex-users (29,153); ^4^ sum of non-users of estrogen-like supplements (20,257) and any type of dietary supplements (31,868).

**Table 3:** Factors associated with estrogen-like supplement use.

	Univariable OR (95% CI)	*p*-Value	Multivariable OR (95% CI)	*p*-Value
**Age**				
10s	Reference		Reference	
20s	1.67 (1.32–2.11)	<0.001	1.52 (1.20–1.93)	<0.001
30s	1.86 (1.48–2.33)	<0.001	1.72 (1.37–2.17)	<0.001
40s	2.79 (2.24–3.48)	<0.001	2.35 (1.88–2.94)	<0.001
50s	3.46 (2.78–4.31)	<0.001	2.87 (2.27–3.64)	<0.001
60s	1.78 (1.39–2.27)	<0.001	2.09 (1.60–2.73)	<0.001
**Gestational condition**				
None	Reference		Reference	
Pregnant	1.11 (0.90–1.37)	0.313	2.10 (1.65–2.67)	<0.001
Lactating	0.58 (0.46–0.73)	<0.001	1.06 (0.8–1.39)	0.656
**Menstruation**				
No	Reference		Reference	
Yes	0.87 (0.81–0.94)	<0.001	0.75 (0.64–0.86)	0.778
**Menstruation-related Problem**				
No	Reference		Reference	
Yes	1.29 (1.20–1.38)	<0.001	1.40 (1.24–1.58)	<0.001
**Dedication for menstruation-related problems**				
No	Reference		Reference	
See a doctor	2.74 (2.32–3.25)	<0.001	2.34 (1.96–2.79)	<0.001
Take medicine	1.71 (1.53–1.91)	<0.001	1.84 (1.63–2.09)	<0.001
Both doctor and medicine	2.20 (1.85–2.62)	<0.001	2.21 (1.84–2.66)	<0.001
**Subjective symptoms suggestive of menopause**				
No	Reference		Reference	
Yes	3.21 (2.98–3.46)	<0.001	2.54 (2.34–2.75)	<0.001

*n* = 61,021.

**Table 4:** Menstruation-related problems among estrogen-like supplement users.

	*n*	%
None	132	19.2
Irregular cycles	291	42.4
Heavy menstrual bleeding	139	20.3
Severe pain during menstruation (abdominal pain, headaches, etc.)	290	42.3
Feeling irritable, depressed, or sleepy before menstruation	320	46.6
Other	33	4.8

*n* = 686.

**Table 5:** Statement of medication for menstruation-related symptoms among estrogen-like supplement users.

	*n*	%
Seeing a doctor for menstruation-related symptoms	126	11.3
Taking medicine for menstruation-related symptoms	233	20.8
Seeing a doctor other than menstruation-related symptoms	141	12.6
Taking medicine other than menstruation-related symptoms	183	16.4
No medication	664	59.4

*n* = 1118, excluding pregnancy and lactating participants.

**Table 6:** Subjective symptoms suggestive of menopause among estrogen-like supplement users.

	*n*	%
I have subjective symptoms and am visiting a clinic	112	10.0
I have subjective symptoms but am not visiting a clinic	326	29.2
I used to have symptoms, but I am better now	196	17.5
I have never experienced any symptoms	484	43.3

*n* = 1118, excluding pregnancy and lactating participants.

**Table 7:** The purpose of estrogen-like supplement use.

	All	10s	20s	30s	40s	50s	60s	*p*-Value
*n*	1200	130	203	217	217	217	216	
Weight loss	20.0	33.8	34.5	25.3	12.9	9.7	10.2	<0.001
Mastogenic effect	15.7	40.0	37.4	15.2	6.5	3.7	2.3	<0.001
Skincare	31.0	34.6	42.9	40.6	28.1	19.8	22.2	<0.001
Treatment for menstruation-related symptoms	24.5	34.6	35.0	39.6	31.3	9.2	1.9	<0.001
Treatment for menopause symptoms	33.5	7.7	12.3	25.3	51.6	66.8	25.5	<0.001
Prevention of diseases	11.4	5.4	7.9	15.2	8.8	12.9	15.7	0.006
Treatment of diseases	4.3	3.8	4.9	6.5	3.2	2.8	4.6	0.470
Anti-aging	36.8	3.8	15.3	38.2	42.4	43.3	63.4	<0.001
No special reason	4.8	3.8	3.9	5.5	5.1	3.7	6.5	0.734
Other	3.1	0.8	3.4	5.1	3.2	2.3	2.8	0.325

*n* = 1200. Multiple answers. The results are shown as percentages (%). The difference among generations was examined using a chi-squared (*χ*^2^) test.

**Table 8:** The major ingredients of estrogen-like supplements.

	All	10s	20s	30s	40s	50s	60s	*p*-Value
*n*	1200	130	203	217	217	217	216	
Soybeans/isoflavones	43.3	28.5	40.9	44.2	45.2	46.5	48.1	0.008
Equol	24.4	3.1	10.3	18.0	34.1	41.0	30.6	<0.001
Red clover	2.9	1.5	6.4	1.8	4.1	1.8	1.4	0.014
Kudzu vine	2.7	2.3	3.9	3.7	2.8	0.9	2.3	0.433
Chaste tree (Chaste berry)	3.4	3.1	4.9	5.5	3.7	2.3	0.9	0.097
Black cohosh	2.4	1.5	3.9	4.6	2.3	1.4	0.5	0.045
*Pueraria mirifica*	5.2	6.9	10.8	6.5	3.7	2.8	1.4	<0.001
Placenta	22.7	13.1	21.2	23.0	23.5	19.4	31.9	0.002
French marine pine bark extract	1.7	0.0	2.5	1.4	2.3	2.8	0.5	0.210
Other	11.1	13.1	7.9	12.9	10.6	12.9	9.7	0.473

*n* = 1200. Multiple answers. The results are shown as percentages (%). The difference among generations was examined using a chi-squared (*χ*^2^) test.

**Table 9:** Frequency of estrogen-like supplement use.

	*n*	%
5–7 days/week	835	69.6
3–4 days/week	146	12.2
1–2 days/week	117	9.8
1–3 days/month	44	3.7
1 day/ few months	37	3.1
Other	21	1.8

*n* = 1200.

**Table 10:** Duration of estrogen-like supplement use.

	*n*	%
Under 1 week	61	5.1
1 week–1 month	129	10.8
1–3 months	213	17.8
3–6 months	172	14.3
7–11 months	142	11.8
More than 1 year	411	34.3
Do not remember	72	6.0

*n* = 1200.

**Table 11:** Information sources of estrogen-like supplements.

	All	10s	20s	30s	40s	50s	60s	*p*-Value
*n*	1200	130	203	217	217	217	216	
Television or radio	26.8	23.1	22.2	27.2	22.6	30.4	33.8	0.035
Newspaper, magazine, or advertisement	16.5	12.3	13.3	15.7	11.5	17.5	26.9	<0.001
Internet	58.5	49.2	50.2	56.7	65.4	66.8	58.3	<0.001
SNS (LINE, Facebook, Twitter, and Instagram)	18.8	34.6	31.5	30.0	12.4	6.5	4.6	<0.001
Specialists (doctors, pharmacists, and dieticians)	10.8	10.8	12.8	11.1	12.9	8.3	8.8	0.523
Beauty salon or therapist	4.2	3.8	9.4	5.1	2.8	1.8	2.3	0.001
Store clerks in pharmacies or drugstores	14.1	16.2	14.3	16.1	15.7	10.6	12.5	0.508
Point-of-purchase adverts	8.3	1.5	7.9	12.0	11.1	9.7	5.1	0.004
Product packaging	16.6	6.9	14.3	19.4	22.1	16.6	16.2	0.008
Family, friends, or acquaintances	19.8	26.9	18.2	19.8	16.6	16.6	23.1	0.116
Other	1.9	0.0	1.0	1.8	2.3	1.8	3.7	0.197

*n* = 1200. Multiple answers. The difference among generations was examined using a chi-square (*χ*^2^) test.

**Table 12:** Ways of obtaining estrogen-like supplements.

	All	10s	20s	30s	40s	50s	60s	*p*-Value
*n*	1200	130	203	217	217	217	216	
Pharmacy or drug store	44.2	52.3	51.2	56.7	39.2	36.4	32.9	<0.001
Supermarket or convenience store	7.5	10.0	14.3	8.3	5.5	2.8	5.6	<0.001
Clinic	6.7	13.1	7.9	8.3	9.2	1.4	2.8	<0.001
Beauty salon	3.8	3.1	7.9	5.5	3.7	0.5	1.9	0.001
Internet	57.5	36.9	45.3	55.3	59.4	72.8	66.2	<0.001
Mail order	9.9	10.0	12.3	11.5	8.3	4.1	13.4	0.018
Family, friends, or acquaintances	2.8	4.6	4.4	5.1	2.3	0.5	0.9	0.011
Other	1.0	0.0	1.0	1.4	0.5	0.9	1.9	0.569

*n* = 1200. Multiple answers. The difference among generations was examined using a chi-square (*χ*^2^) test.

**Table 13:** Adverse events that might be associated with estrogen-like supplement use.

	*n*	%	Stopped Immediately	Decreased Amount/Frequency	Kept Using
None	1004	83.7			
Nausea or vomiting	56	4.7	42.9	37.5	19.6
Headaches	32	2.7	46.9	37.5	15.6
Diarrhea or constipation	43	3.6	37.2	44.2	18.6
Eczema or itching	18	1.5	55.6	38.9	5.6
Hot flash	28	2.3	35.7	39.3	25.0
Dizzy	22	1.8	40.9	36.4	22.7
Fatigue	23	1.9	43.5	39.1	17.4
Palpitations or shortness of breath	12	1.0	66.7	33.3	0.0
Irregular vaginal bleeding	20	1.7	65.0	25.0	10.0
Breast swelling and pain	31	2.6	29.0	22.6	48.4
Heavy menstruation	28	2.3	32.1	39.3	28.6
Other	11	0.9	18.2	18.2	63.6

*n* = 1200. Multiple answers.

## Data Availability

The data presented in this study are available upon request from the corresponding author.

## Supplementary Materials

The following supporting information can be downloaded at: https://www.mdpi.com/article/10.3390/nu14214509/s1, Supplementary Materials.
