# Supplementary material for: The Prevalence of Dietary Supplements That Claim Estrogen-like Effects in Japanese Women
Source: Nutrients. 2022 Oct 26;14(21):4509. doi: 10.3390/nu14214509 (PMC9653890; doi:10.3390/nu14214509)
Supplement: Supplementary file 1 [file nutrients-14-04509-s001.zip › nutrients-1978078-supplementary.pdf]

Questionnaire

<u>Preliminary survey</u>

SC1 Are you currently pregnant or breastfeeding?

1. Neither.
2. I am currently pregnant.
3. I am currently breastfeeding.

SC2 Are you currently menopausal? If you have gone through menopause, please also indicate the age at which you went through menopause.

1. in 30s
2. in 40s
3. in 50s
4. in 60s
5. Premenopausal
6. Before first menstruation

SC3 Do you currently have symptoms that bother you about menstruation?

1. None
2. Irregular cycles
3. Heavy menstrual bleeding
4. Severe pain during menstruation (abdominal pain, headaches, etc.)
5. Feeling irritable, depressed, or sleepy before menstruation
6. Other

SC4 Are you currently seeing a doctor or taking any medication?

1. Seeing a doctor for menstruation-related symptoms
2. Taking medicine for menstruation-related symptoms
3. Seeing a doctor other than menstruation-related symptoms
4. Taking medicine other than menstruation-related symptoms
5. No medication

SC5 Do you currently have symptoms suggestive of menopause (hot flashes, anxiety, irritability, dizziness, stiff shoulders, fatigue, headaches, etc.)?

1. I have subjective symptoms and am visiting a clinic
2. I have subjective symptoms but am not visiting a clinic
3. I used to have symptoms, but I'm better now
4. I have never experienced any symptoms

SC6 Are you currently using dietary supplements?

1. Yes, I am currently using dietary supplements.
2. No, I am not, but I used to use dietary supplements previously.
3. No, I have never used dietary supplements.

SC7 Have you ever used supplements that claim female hormone-like effects?

1. I am currently using it.
2. I used to use it within the last year.
3. I used to use it over a year ago.
4. I have never used it.

Actual survey

Q1 What purpose are you using, or have you been using, estrogen-like supplements for?

1. Weight loss
2. Mastogenic effect
3. Skincare
4. Treatment for menstruation-related symptoms
5. Treatment for menopause symptoms
6. Prevention of diseases
7. Treatment of diseases
8. Anti-aging
9. No special reason
10. Other

Q2 Which is the major ingredients of estrogen-like supplements that you use/used?

1. Soybeans/isoflavones
2. Equol
3. Red clover
4. Kudzu vine
5. Chaste tree (Chaste berry)
6. Black cohosh
7. Pueraria mirifica
8. Placenta
9. French marine pine bark extract
10. Others

Q3 How often do you use or have you used estrogen-like supplements?

1. 5–7 days/week
2. 3–4 days/week
3. 1–2 days/week
4. 1–3 days/month
5. 1 day/few months
6. Other

Q4 How long have you been or have you been using estrogen-like supplements?

1. Within 1 week
2. 1 week – 1 month
3. 1–3 months
4. 3–6 months
5. 7–11 months
6. More than 1 year
7. Do not remember

Q5 Where do you mainly get information about estrogen-like supplements?

1. Television, radio
2. Newspaper, magazine, advertisement
3. Internet
4. SNS (LINE, Facebook, Twitter, Instagram)
5. Specialists (doctors, pharmacists, dieticians)
6. Beauty salon, therapist
7. Store clerks in pharmacies or drugstores
8. Point-of-purchase adverts
9. Product packaging
10. Family, friends, acquaintances
11. Other

Q6 How do you purchase/obtain or have you purchased/obtained estrogen-like supplements?

1. Pharmacy, drug store
2. Supermarket, convenience store
3. Clinic
4. Beauty salon
5. Internet
6. Mail order
7. Family, friends, acquaintances
8. Other

Q7 Have you ever experienced adverse events associated with estrogen-like supplement use?

1. None
2. Nausea, vomiting
3. Headaches
4. Diarrhea, constipation
5. Eczema, itching
6. Hot flash
7. Dizzy
8. Fatigue
9. Palpitations, shortness of breath
10. Irregular vaginal bleeding
11. Breast swelling and pain
12. Heavy menstruation
13. Other

Q8 Did you stop using the product when you experienced each adverse event after using estrogen-like supplement?

1. Stopped im-mediately
2. Keep using with decreased amount/frequency
3. Kept using

Thank you for your cooperation
